# Magnetoencephalography detects phase-amplitude coupling in Parkinson’s disease

**DOI:** 10.1038/s41598-022-05901-9

**Published:** 2022-02-03

**Authors:** Masataka Tanaka, Takufumi Yanagisawa, Ryohei Fukuma, Naoki Tani, Satoru Oshino, Masahito Mihara, Noriaki Hattori, Yuta Kajiyama, Ryota Hashimoto, Manabu Ikeda, Hideki Mochizuki, Haruhiko Kishima

**Affiliations:** 1 Department of Neurosurgery, Osaka University Graduate School of Medicine, 2-2 Yamadaoka, Suita, Osaka 565-0871 Japan; 2 Institute for Advanced Co-Creation Studies, Osaka University, 2-2 Yamadaoka, Suita, Osaka 565-0871 Japan; 3 Department of Neuroinformatics, ATR Computational Neuroscience Laboratories, 2-2-2 Hikaridai, Seika-cho, Kyoto, 619 0288 Japan; 4 Department of Neurology, Osaka University Graduate School of Medicine, 2-2 Yamadaoka, Suita, Osaka 565-0871 Japan; 5 Department of Rehabilitation, Faculty of Medicine, Academic Assembly, University of Toyama, Toyama, Japan; 6 Department of Pathology of Mental Diseases, National Institute of Mental Health, National Center of Neurology and Psychiatry, 4-1 Ogawahigashi, Kodaira, Tokyo 187-8553 Japan; 7 Department of Psychiatry, Osaka University Graduate School of Medicine, 2-2 Yamadaoka, Suita, Osaka 565-0871 Japan; 8 Molecular Research Center for Children’s Mental Development, United Graduate School of Child Development, Osaka University, 2-2 Yamadaoka, Suita, Osaka 565-0871 Japan

**Keywords:** Neuroscience, Neurology

## Abstract

To characterize Parkinson’s disease, abnormal phase-amplitude coupling is assessed in the cortico-basal circuit using invasive recordings. It is unknown whether the same phenomenon might be found in regions other than the cortico-basal ganglia circuit. We hypothesized that using magnetoencephalography to assess phase-amplitude coupling in the whole brain can characterize Parkinson’s disease. We recorded resting-state magnetoencephalographic signals in patients with Parkinson’s disease and in healthy age- and sex-matched participants. We compared whole-brain signals from the two groups, evaluating the power spectra of 3 frequency bands (alpha, 8–12 Hz; beta, 13–25 Hz; gamma, 50–100 Hz) and the coupling between gamma amplitude and alpha or beta phases. Patients with Parkinson’s disease showed significant beta–gamma phase-amplitude coupling that was widely distributed in the sensorimotor, occipital, and temporal cortices; healthy participants showed such coupling only in parts of the somatosensory and temporal cortices. Moreover, beta- and gamma-band power differed significantly between participants in the two groups (*P* < 0.05). Finally, beta–gamma phase-amplitude coupling in the sensorimotor cortices correlated significantly with motor symptoms of Parkinson’s disease (*P* < 0.05); beta- and gamma-band power did not. We thus demonstrated that beta–gamma phase-amplitude coupling in the resting state characterizes Parkinson’s disease.

## Introduction

Dysrhythmia contributes to the motor symptoms of Parkinson’s disease. In previous studies, patients with Parkinson’s disease who were in a resting state were observed to have abnormal synchronization in cortico-basal ganglia circuits, including the subthalamic nucleus, globus pallidus internus, and primary motor cortex^1,2^. Beta oscillations in the subthalamic nucleus have been correlated with motor symptoms, such as bradykinesia and rigidity^3,4^, although recent evidence has been contradictory^5^. Excessive beta oscillations in the cortico-basal ganglia circuit have been shown to represent pathologic oscillations in Parkinson’s disease.

The beta oscillation phase measured in patients with Parkinson’s disease during the resting state has been significantly coupled with the gamma oscillation (> 50 Hz) amplitude in the subthalamic nucleus^6,7^ and motor cortex^8,9^; accordingly, it has been termed beta–gamma phase-amplitude coupling (PAC)^10,11^. Cortical beta–gamma PAC has been correlated with Parkinsonian motor symptoms and is attenuated by deep brain stimulation and levodopa^12^. During movement, PAC was attenuated earlier in patients with Parkinson’s disease than in study participants without movement disorders^9^. Excessive beta oscillations and beta–gamma PAC in the cortico-basal ganglia circuit are therefore potential cortical biomarkers of Parkinsonian motor symptoms^13^.

Previous evaluations of PAC were mostly conducted in the cortico-basal ganglia circuit and used intracranial electrodes, such as electrodes for deep brain stimulation, or used subdural electrodes on the sensorimotor cortex to measure electrocorticographic signals. Whether the PAC of gamma oscillation in patients with Parkinson’s disease differs from that in healthy study participants (HSPs) of similar ages has not been well examined. Although some studies using electroencephalography revealed exaggerated PAC in Parkinson’s disease, anatomic differences in PAC have not been compared between patients and HSPs because of the low spatial resolution of electroencephalography^14,15^. However, recent studies have shown that magnetoencephalography (MEG) can evaluate cortical PAC with high reliability^14–16^. We therefore recorded MEG signals to evaluate PAC during the resting state both in patients with Parkinson’s disease and in age-matched HSPs. We hypothesized that beta–gamma PAC estimated from MEG signals could be used to characterize patients with Parkinson’s disease and thus serve as a biomarker for Parkinsonian motor symptoms.

## Results

### Participants

Between May 2015 and February 2018, 32 patients with Parkinson’s disease and 54 HSPs were recruited at Osaka University Hospital in Japan. Before data analysis, 9 patients and 17 HSPs were excluded: 2 patients and 5 HSPs were excluded because they fell asleep or moved during the resting-state recording; 2 patients and 7 HSPs were excluded because of metal artifact contamination; 1 patient and 5 HSPs were excluded because of equipment trouble; and 4 patients were excluded because of another movement disorder (essential tremor) or motor impairment (limb paresis caused by polio) and a change in diagnosis to multiple system atrophy. The subsequent analyses included the remaining 23 patients with Parkinson’s disease (11 men; mean age: 65.3 ± 7.9 years; Table 1) and 23 of the remaining 37 HSPs who were age- and sex-matched to the patients (11 men; mean age: 62.8 ± 5.7 years).

**Table 1:** Clinical characteristics of the participants with Parkinson’s disease.

Patients		Age (years)	More affected side	MDS-UPDRS-III akinesia score	On/off state during recording	LEDD (mg/dL)	Disease duration (years)
ID	Sex						
1	Female	68	Right	8	On	625	5
2	Female	76	Right	5	On	0	1
3	Male	73	Left	13	On	0	4
4	Male	54	Left	5	On	340	3
5	Female	72	Left	4	On	37.5	30
6	Female	69	Left	7	On	175	4
7	Male	75	Left	17	On	400	8
8	Male	69	Left	12	Off	1406	15
9	Female	70	Left	9	On	891.5	10
10	Female	52	Left	1	On	715	11
11	Female	72	Left	15	On	100	1
12	Male	69	Left	12	On	110	4
13	Male	54	Right	14	Off	1344.5	11
14	Female	68	Right	7	On	100	4
15	Male	63	Left	19	On	1037.5	7
16	Female	55	Right	20	Off	560	6
17	Male	64	Left	12	On	0	3
18	Female	69	Left	8	On	200	5
19	Female	72	Left	14	On	975	18
20	Male	52	Left	13	On	549	4
21	Male	53	Right	11	On	1081	18
22	Male	66	Left	14	On	752.2	8
23	Female	66	Left	21	Off	948	5

*MDS-UPDRS-III akinesia score* sum of akinesia-related scores on the Movement Disorder Society–sponsored revision of the Unified Parkinson’s Disease Rating Scale, part III; *LEDD* levodopa equivalent daily dose.

### Beta–gamma PAC characterizes Parkinson’s disease

**Figure 1:**
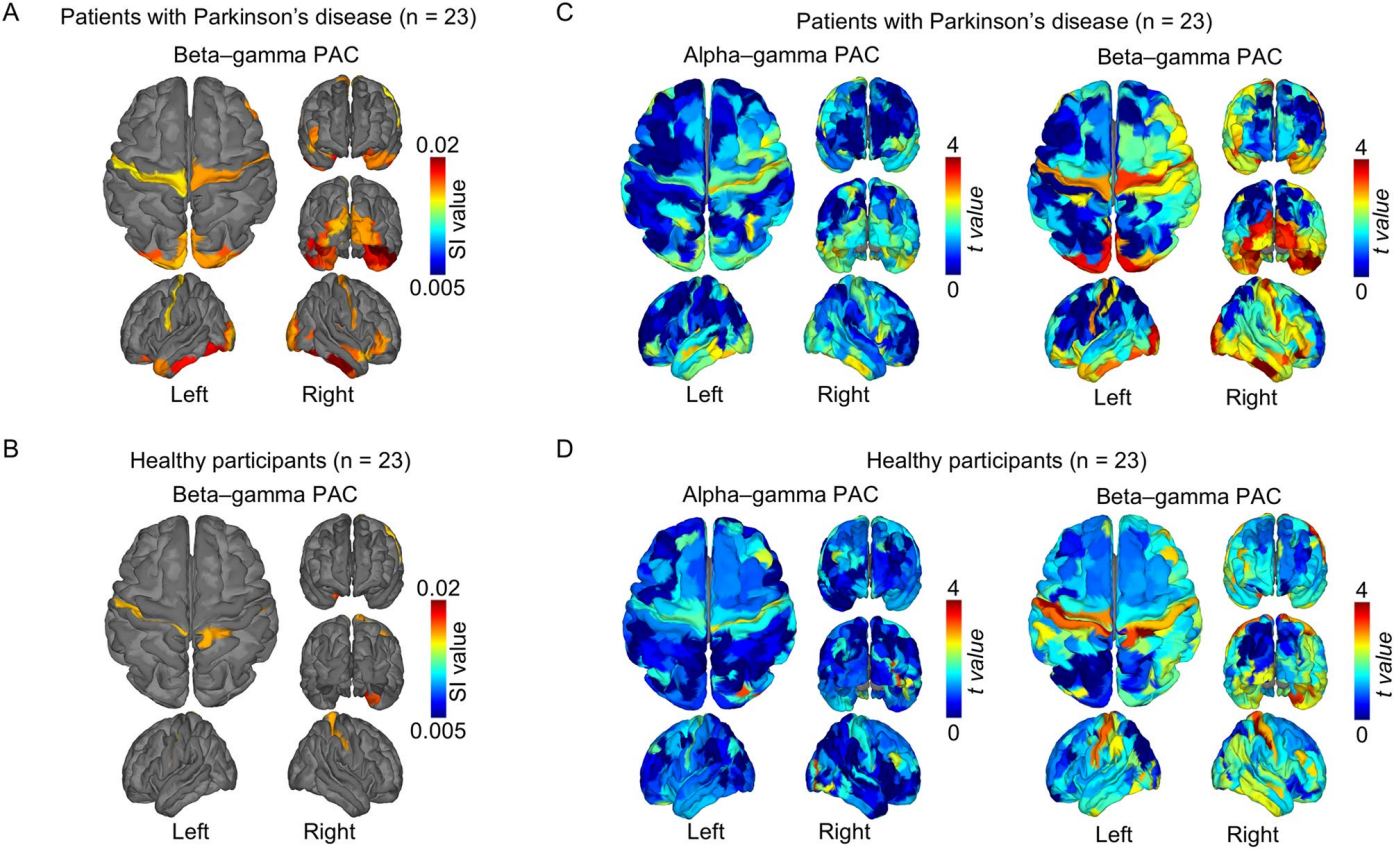
Beta–gamma phase-amplitude coupling during the resting state. For each study group (patients with Parkinson’s disease and healthy study participants), we averaged synchronization index (SI) values for beta–gamma coupling and color-coded them on the cortex of (**A**) patients (n = 23) and (**B**) healthy participants (n = 23). The SI values are shown only if they significantly exceeded the corresponding phase-shuffled values in (A) and (B). The *t value*s between SI values and phase-shuffled SI values are color-coded on the cortex of (**C**) patients (n = 23) and (**D**) healthy participants (n = 23) for alpha–gamma coupling and beta–gamma coupling.

For each participant, MEG signals obtained during the resting state were used to estimate the cortical currents at each cortical point (vertex) on the FreeSurfer atlas^17^. Noisy MEG segments with artifacts, such as muscle activity, were excluded from analysis during the estimation (see Supplementary Table S1 for the data length after removal of noisy signals). Then, for the estimated cortical currents, we evaluated PAC during the resting state using a synchronization index (SI)^18^ for gamma-band (50–100 Hz) amplitude and alpha- (8–12 Hz) or beta-band (13–25 Hz) phases at each cortical vertex. The SI values for alpha–gamma PAC and beta–gamma PAC were averaged at each of 360 cortical areas in Human Connectome Project (HCP) parcellation^19^ for all patients and HSPs. Then, within each participant group, the estimated PACs were tested to determine whether they were significantly higher than the phase-shuffled SI values, thus showing the significance of the estimated PAC. For the 360 cortical areas in patients with Parkinson’s disease, SI values for beta–gamma PAC were significantly higher than the phase-shuffled SI values in the sensorimotor, occipital, and temporal cortices (Fig. 1A; *P* < 0.05, 2-tailed paired Welch *t test*, false discovery rate corrected; also see Supplementary Table S2 for details of the areas with significant beta–gamma PAC). In contrast, in HSPs, significant beta–gamma PACs were observed in parts of the somatosensory and temporal cortices (Fig. 1B). Significant beta–gamma PAC during the resting state was noninvasively observed to be more widespread in patients with Parkinson’s disease than in HSPs. In contrast, no significant alpha–gamma PAC during the resting state was observed in either patients with Parkinson’s disease or HSPs, although the SI values for alpha–gamma PAC tended to be higher in the sensorimotor and temporal cortices compared to the phase-shuffled SI values (Fig. 1C,D). In addition to assessing the significance of the SI values within the participant groups, we compared participants’ SI values directly between the groups. However, in a direct comparison of participants in the two groups, no significant differences in SI values were evident for either alpha–gamma PAC or beta–gamma PAC (*P* > 0.05, *F test*, clusterwise corrected). These results demonstrate significant beta-gamma PAC in the sensorimotor cortex in participants in both groups and in the occipito-temporal cortex for patients with Parkinson's disease, although the differences between groups were not significant.

### Representative results of PAC and power from one participant

**Figure 2:**
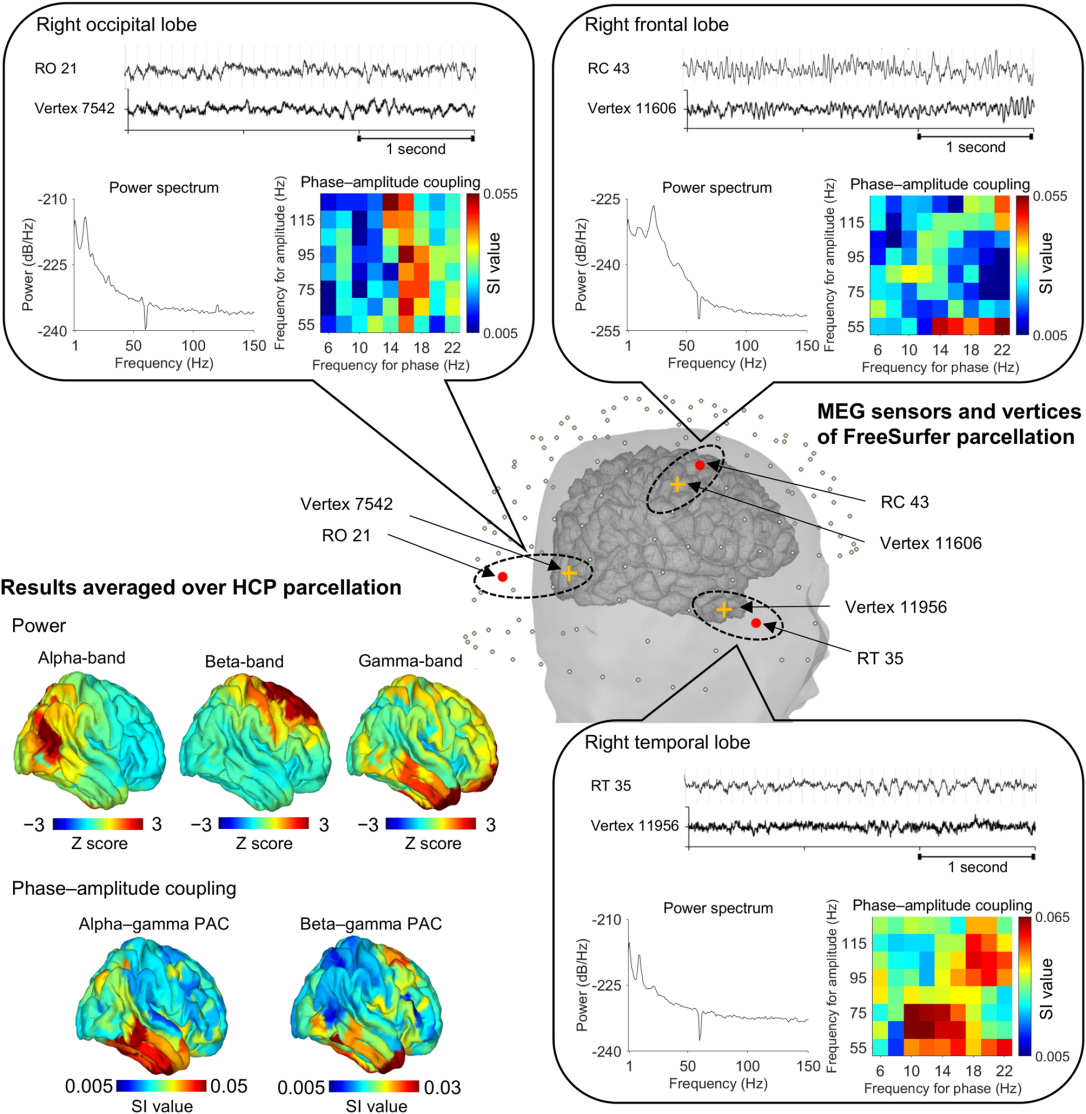
Representative MEG signals. This sample analysis shows the phase-amplitude coupling (PAC) and power calculated from data for patient #21. In the center, the scalp and the extracted cortex are superimposed, with magnetoencephalography (MEG) sensors overlaid. Three cortical vertices (crosses) were selected from FreeSurfer parcellation. The MEG sensors close to them (circles) were also selected. Signals from the selected MEG sensors and estimated cortical currents for the selected vertices are shown. Power spectra and synchronization index (SI) values calculated from each cortical current are plotted. In the SI plots, each phase has a 4-Hz frequency range (that is, “6 Hz” in frequency for phase corresponds to “4–8 Hz”), and each frequency range has a 50-Hz amplitude range (that is, “55 Hz” in frequency for amplitude corresponds to “30–80 Hz”). At the lower left, Z scores for power and SI values calculated on vertices of the right hemisphere are grouped according to Human Connectome Project parcellation, averaged in each cortical area and color-coded on the cortex. This figure was created using Brainstorm (http://neuroimage.usc.edu/brainstorm) and MATLAB R2015b (MathWorks, Natick, Massachusetts, USA).

After obtaining the results of significant beta–gamma PAC, we further examined the cortical currents at three cortical areas of HCP parcellation with high beta–gamma PAC: sensorimotor, occipital and temporal cortices. First, we examined the raw MEG signals and the estimated cortical currents to confirm that the estimated high PAC did not originate from artifacts, such as muscle activity, although we removed such artifacts during signal preprocessing (see Methods). Figure 2 shows representative results from 1 participant (patient #21). Both the MEG signals and the estimated cortical currents had no apparent muscle artifacts and were different among the three cortical points in their characteristic frequency properties. For each cortical point, the power spectrum density of the estimated cortical currents demonstrated that they have different characteristic frequencies. The cortical currents at the motor cortex showed high beta-band power, while the cortical currents at the occipital and temporal cortices showed high alpha-band power. When we calculated the power of 3 frequency bands (alpha [8–12 Hz], beta [13–25 Hz] and gamma [50–100 Hz]) for all vertices, with normalization across the entire brain to obtain Z scores, the Z scores for each frequency band were heightened at different cortical areas: that for alpha-band power was heightened in the occipital cortex, that for beta-band power was heightened in the frontal cortex, and that for gamma-band power was heightened in the temporal cortex. Then, for each cortical point, we evaluated the SI values for various combinations of the frequency bands. Strong coupling between gamma-band amplitude and alpha- or beta-band phases was observed at the vertex of the temporal cortex. When we estimated SI values for all cortical vertices and color-coded the SI values on the cortex, the alpha–gamma and beta–gamma couplings were high at the temporal cortex for this representative patient. Anatomic differences in PAC were confirmed by step-by-step analysis of the cortical currents estimated from MEG signals for this representative patient.

### Significant PAC in representative cortical areas

**Figure 3:**
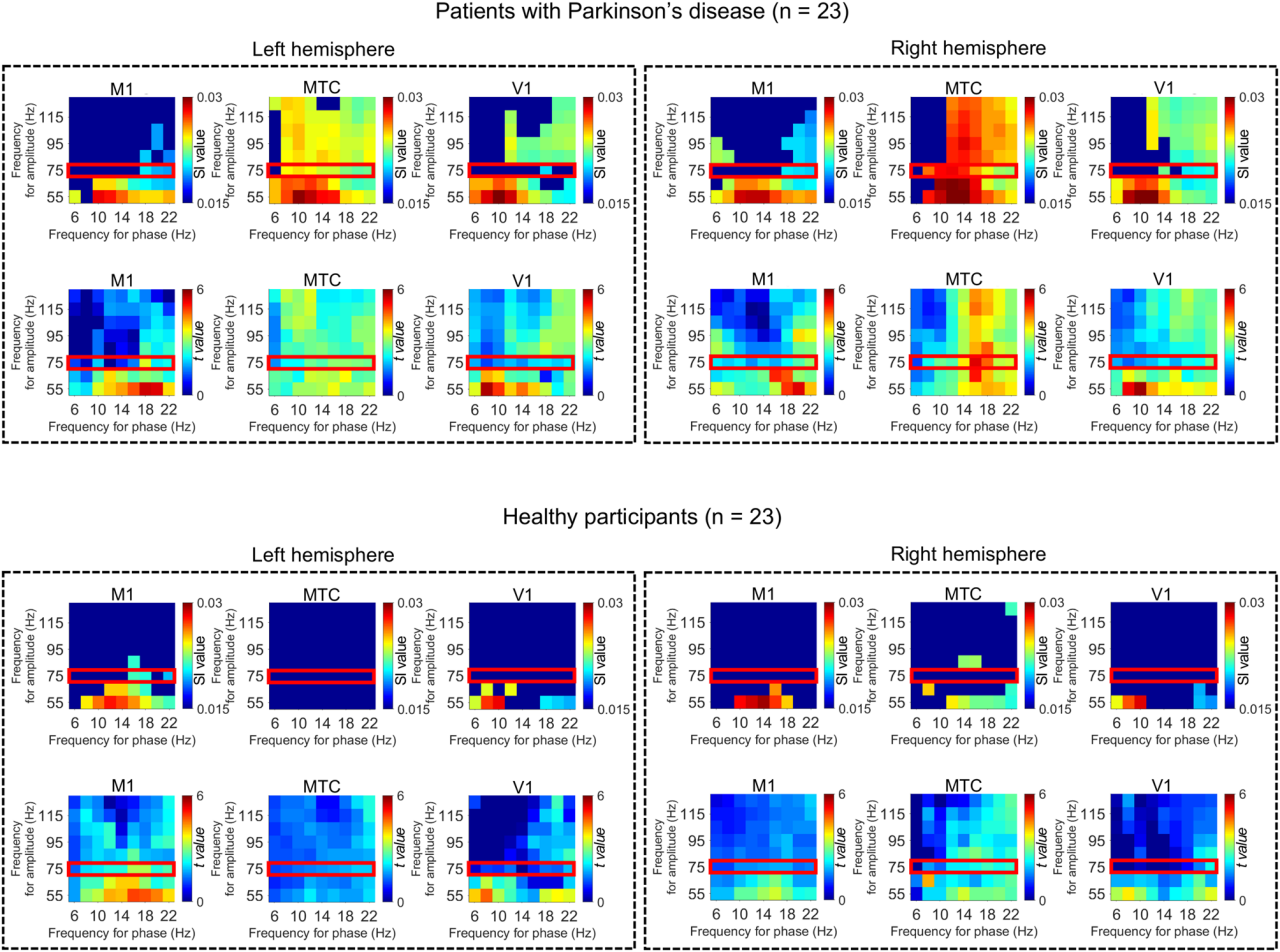
Phase–amplitude coupling at representative cortical areas. The synchronization index (SI) values for various combinations of frequency bands were calculated at cortical vertices belonging to representative cortical areas and averaged for each cortical area. The primary motor cortex (M1, Human Connectome Project [HCP] parcel #8), the anterior middle temporal cortex (MTC, HCP parcel #134), and the primary visual cortex (V1, HCP parcel #1) of both hemispheres were selected as representative cortical areas. The SI values were averaged for each participant group, and only SI values that significantly exceeded the phase-shuffled SI values were plotted (*P* < 0.05, 2-tailed paired Welch *t test*, false discovery rate corrected). Each phase has a 4 Hz frequency range (that is, “6 Hz” in frequency for phase corresponds to “4–8 Hz”), and each amplitude has a 50 Hz frequency range (that is, “55 Hz” in frequency for amplitude corresponds to “30–80 Hz”). The corresponding *t value*s are shown below each of the SI values. The red squares indicate the SI values for gamma amplitude (50–100 Hz).

For the three selected cortical areas of all patients and HSPs, we evaluated the SI values for various combinations of frequency bands for phase and amplitude. The SI values were averaged for each cortical area to demonstrate the characteristic PAC for each area. The averaged SI values were compared with the phase-shuffled SI values to show the statistical significance within each participant group. The SI values at the three selected cortical areas demonstrated significantly high alpha–gamma PAC and beta–gamma PAC in patients with Parkinson's disease (Fig. 3). In contrast, the SI values for gamma amplitude (red square in Fig. 3) in HSPs were significant only for beta–gamma PACs in the left motor cortex. These results are consistent with the results shown in Fig. 1B,D. In addition, the SI values were significant for more combinations of phases and amplitudes in patients with Parkinson's disease compared to those of HSPs. It should be noted that although alpha–gamma PACs were significant in the temporal cortex, their *t value*s tended to be lower than those of beta–gamma PACs, which is consistent with the results shown in Fig. 1A,C.

### Cortical power differed between the participant groups

**Figure 4:**
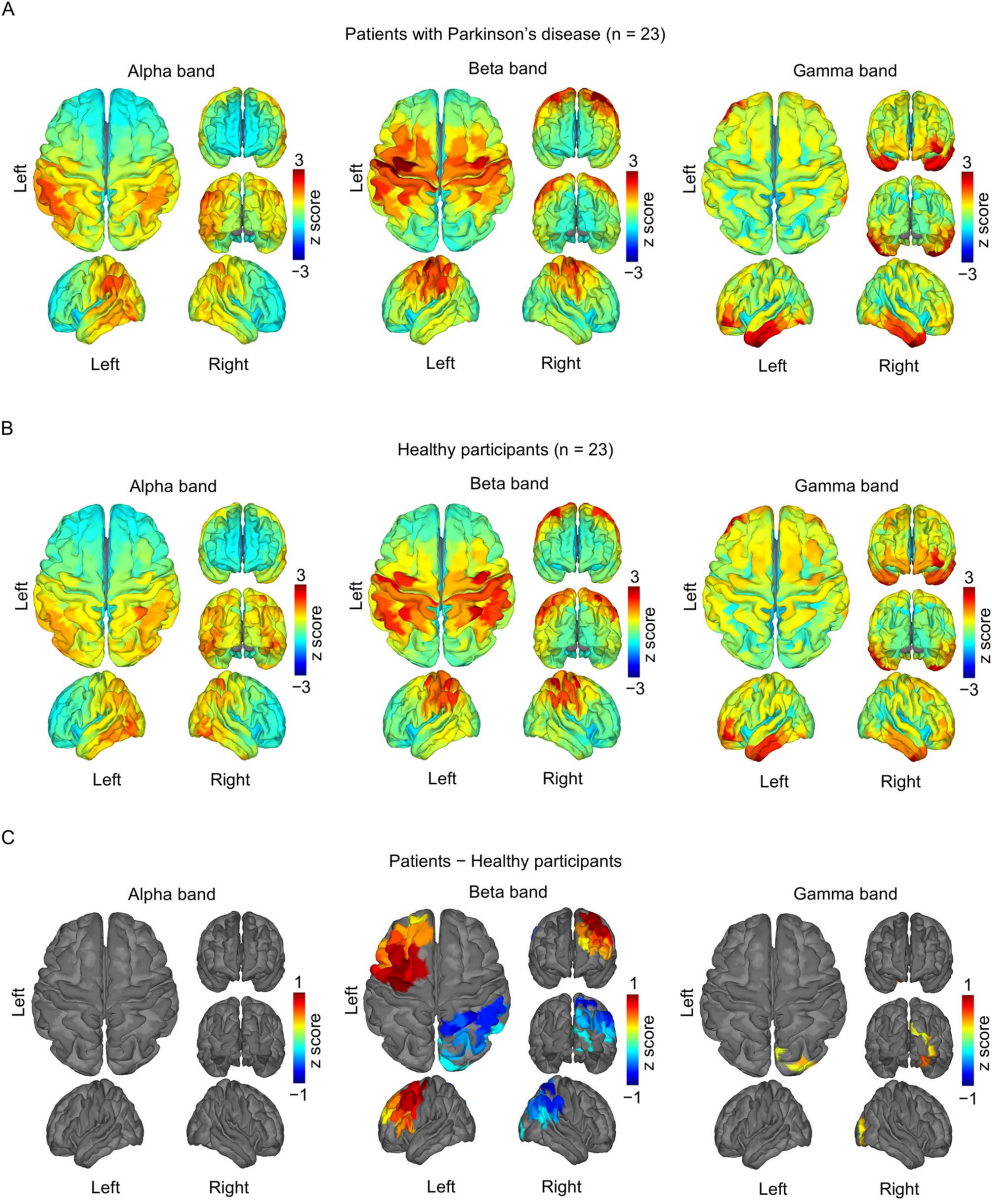
Cortical power during the resting state. The normalized power of 3 frequency bands (alpha, beta, and gamma) was averaged for (**A**) the patients with Parkinson's disease (n = 23) and (**B**) the healthy study participants (n = 23) and was color-coded for each cortical area. (**C**) The differences in mean power between the participant groups (**A**,**B**) were color-coded for the participants’ cortical areas with statistically significant differences.

The cortical power of 3 frequency bands (alpha, beta, and gamma) was also evaluated at each cortical area (Fig. 4A,B) and was compared for the two participant groups across the entire brain. For both groups, alpha-band power was evident in the participants’ parieto-occipital cortex, beta-band power was evident in the participants’ sensorimotor cortex, and gamma-band power was higher in the participants’ fronto-temporal cortex. In patients with Parkinson's disease (compared with HSPs), beta-band power in the left frontal cortex and gamma-band power in the right occipital cortex were significantly increased and beta-band power in the right parietal cortex was decreased that of compared to HSPs (Fig. 4C; *P* < 0.05, *F test*, clusterwise corrected). It should be noted that the difference in beta- or gamma-band power between the patients with Parkinson's disease and HSPs did not explain the different patterns of beta–gamma PAC in participants in the two groups.

### Phases for each frequency band did not differ between the participant groups

To assess the contributions of phase to the various frequency bands, we defined phase values for lower-frequency signals and higher-frequency amplitude filtered by the lower-frequency signals. “Phase value” was defined as the absolute value of a time averaged complex exponential for the analytic phase. The phase values of each frequency component on each cortical area were averaged and compared between the participant groups. Phase values in the two groups showed no significant difference (Supplementary Figure S1). It should be noted that the difference in beta- or gamma-band phase between the patients with Parkinson's disease and HSPs did not explain the different patterns of beta–gamma PAC in participants in the two groups.

### Beta–gamma PAC correlates with motor symptoms of Parkinson’s disease

**Figure 5:**
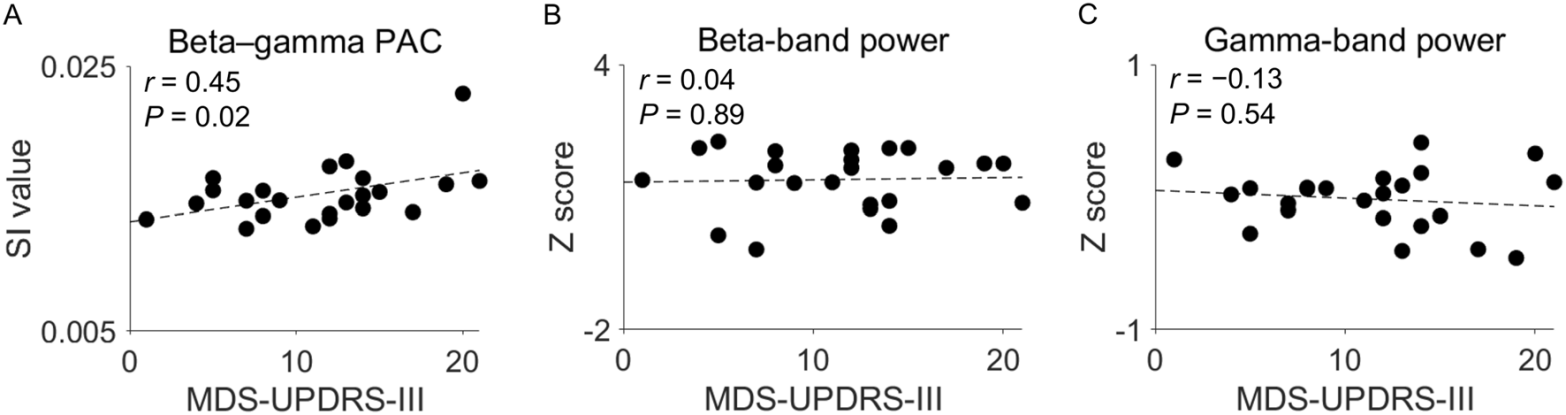
Correlations of disease symptoms with beta–gamma phase–amplitude coupling (PAC) and with beta-band or gamma-band power in the sensorimotor cortex. (**A**) The averaged synchronization index (SI) values for the sensorimotor cortex, in which significant PAC is present, were plotted against the sum of the MDS-UPDRS-III scores for akinesia. (**B**,**C**) The averaged Z scores of beta-band power (**B**) and gamma-band power (**C**) in the sensorimotor cortex were plotted against the sum of the MDS-UPDRS-III scores for akinesia. In the plots, each dot represents a patient with Parkinson’s disease (n = 23); dashed least-squares lines are also shown. MDS-UPDRS-III = Movement Disorder Society–sponsored revision of the Unified Parkinson’s Disease Rating Scale, part III.

Last, we evaluated the correlation between beta–gamma PAC in the sensorimotor cortices and akinesia as scored using part III of the Movement Disorder Society–sponsored revision of the Unified Parkinson’s Disease Rating Scale (MDS-UPDRS-III) for patients with Parkinson’s disease (Fig. 5). The averaged SI values of beta–gamma PAC in the early somatosensory and motor cortices, in which significant beta–gamma PAC was present, correlated with the MDS-UPDRS-III scores for akinesia (Pearson correlation coefficient: *r* = 0.45, *P* = 0.02; Fig. 5A). However, the averaged Z scores of beta-band power in the same area did not correlate with the MDS-UPDRS-III scores for akinesia (*r* = 0.04, *P* = 0.89; Fig. 5B), nor did those of gamma-band power (*r* =  − 0.13, *P* = 0.54; Fig. 5C). In addition, neither the SI values of beta–gamma PAC nor beta-band power were significantly correlated with the MDS-UPDRS-III scores for akinesia in the temporal and occipital cortices, where beta–gamma PACs were significant in patients with Parkinson's disease, as shown in Fig. 1A, and in the left frontal cortex, where the patients had significantly higher beta-band power than HSPs, as shown in Fig. 4C (Supplementary Figure S2).

## Discussion

By evaluating MEG signals in the resting state, we demonstrated that the motor symptoms of Parkinson’s disease as evaluated by MDS-UPDRS-III scores are significantly correlated with resting-state beta–gamma PAC in the sensorimotor cortex, independent of beta-band power. These results are consistent with findings in previous studies that used invasive measurement techniques, which suggested that during the resting state, beta–gamma PAC in the precentral gyrus is a biomarker of Parkinson’s disease^8^. Although we observed no significant difference in SI values during direct comparisons of patients and HSPs, the presence of significant beta–gamma PAC in the sensorimotor, occipital, and temporal cortices characterized patients with Parkinson’s disease. Using noninvasive MEG signals, we have demonstrated that beta–gamma PAC during the resting state is a biomarker predicting motor symptoms in Parkinson’s disease.

We also detected significant beta–gamma PAC at the occipital and temporal cortices in patients. To our knowledge, our report is the first to show significant beta–gamma PAC in the occipito-temporal cortex in patients with Parkinson’s disease. Beta–gamma PAC in the visual cortex might have pathologic significance; moreover, it might be associated with impaired visual perception^20–22^ and with an accumulation of Lewy bodies in the temporal lobe^23^. Although we mainly used MDS-UPDRS-III scores to evaluate the patients’ symptoms, further study to evaluate cognitive and visual perceptual abilities of patients with Parkinson's disease will be necessary to assess the relationship between occipito-temporal PAC and symptoms.

Although our results suggest that significant beta–gamma PAC is a biomarker of Parkinson's disease, we observed no difference in the SI values across the entire brain for beta–gamma PAC in a direct comparison between patients with Parkinson's disease and HSPs. This finding might be related to patients being allowed to take antiparkinsonian agents and to most of the patients being in the ON state during MEG recording, which could cause abnormal PAC to be suppressed. However, the fact that the SI values for beta–gamma PAC in the sensorimotor cortex nevertheless correlated with motor symptoms means that beta–gamma PAC is a biomarker that reflects symptoms even if patients continue to take medication. Thus, even if our current data do not lead to the use of PAC for the classification of patients and healthy participants, PAC can be used in clinical practice as an objective biomarker of motor symptoms.

Characterization of PAC by SI values requires meticulous interpretation. Some previous studies demonstrated that instantaneous, filtering- and Hilbert-based methods to evaluate PAC can potentially lead to spurious estimates of the relation between phase and amplitude^24–26^. For the local field potential (LFP) in the subthalamic nucleus of Parkinson's disease, the nonsinusoidally sharp waveform in the LFP leads to high estimated PAC values given the relation between instantaneous phase and amplitude^27^. Such a sharp waveform is the characteristic of the waveform of Parkinson’s disease. Therefore, even when high SI values relate to the characteristic waveform, high SI values indicate Parkinson’s disease. Although an evaluation of whether the evaluated SI values represent the true PAC is valuable, as Aru et al. discussed in their review of PAC, the evaluated PAC values are useful as a biomarker even when they have the characteristics of a nonsinusoidal waveform^28^.

Notably, the observed PAC for alpha–gamma coupling (Fig. 3) showed SI values that were significantly larger than the phase-shuffled SI values. In addition, the SI values for alpha–gamma coupling were larger than those for beta–gamma coupling in the frontal and occipital cortices, especially for couplings with low gamma-band amplitude (Fig. 3). These data are consistent with the findings of previous studies showing significant alpha–gamma coupling in the sensorimotor cortex^11,29^ and in the visual cortex^30^. However, in the comparison across participants’ entire brains, no significance for alpha–gamma coupling was found (*P* > 0.05, false discovery rate corrected), which is not surprising given the low *t value*s for SI values for alpha–gamma PAC. Therefore, beta–gamma PAC, and not alpha–gamma PAC, is suggested to be a biomarker of Parkinson's disease.

Concerns might arise that the strong beta–gamma PAC in the temporal region is related to muscle activity, motion, and muscle artifacts. As shown in Fig. 2 and confirmed by the authors, such artifacts were not observed in the data used in the analysis after denoising and excluding the remaining noisy segments. Additionally, as shown in Supplementary Figure S3, the correlation between PAC and power was much higher in the frontal cortex than in the temporal cortex, which is inconsistent with the expectation that the correlation should be higher in the temporal cortex if PAC is influenced by high-frequency muscle activity. These results suggest that high and significant PAC was not related to artifacts.

One might argue that the observed beta–gamma PAC in the occipito-temporal cortex was reflected by the activity in the basal ganglia, which might project to the cortex. Basically, deep brain signals detected by MEG affect the wide cortical areas. The high beta–gamma PAC in the occipito-temporal cortex could be attributed to activity in the basal ganglia. However, it is difficult to explain the high beta–gamma PAC in the sensorimotor cortex, which differs from the occipito-temporal cortex, because the MEG signals were detected continuously in the space. In addition, the activity in the basal ganglia, whose neurons are not spatially aligned, have difficulty generating large signals that are detectable by MEG outside of the head. Therefore, it is difficult to attribute the significant beta–gamma PAC in the sensorimotor cortex and occipito-temporal cortex to the activity in the basal ganglia.

Our findings show that beta- and gamma-band power differed significantly for patients with Parkinson's disease and for HSPs. With respect to using MEG to evaluate cortical power in Parkinson's disease, although one study concluded that beta-band power in the primary motor cortex is reduced in patients with Parkinson’s disease compared with that of control participants^31^, another study found increased beta-band power in the sensorimotor cortex in patients with Parkinson's disease compared with that of control participants^32^. In contrast, in studies using scalp electroencephalography, no significant differences in beta-band power were observed in patients with Parkinson’s disease compared with that of HSPs^33–35^. Thus, the results from previous studies of cortical power in Parkinson's disease are not consistent. Moreover, one report suggested that dopamine replacement therapy increases beta-band power in the motor cortex^31^. Cortical power is thought to be strongly influenced by dopamine replacement therapy^36^, complicating the interpretation of the results for cortical power in our study, which was conducted while patients continued their medication.

In previous studies that evaluated patients with Parkinson’s disease during the resting state by scalp electroencephalography, electrocorticography, and MEG, cortical beta-band power was reported not to correlate with clinical motor scores^37^, to vary independently of changes in motor symptoms^12^, and not to differ between on and off cycles^33,34^. In our study, beta-band power in the sensorimotor cortex did not significantly explain Parkinsonian motor symptoms, which was consistent with findings in previous studies.

Although our method was successful in identifying beta–gamma PAC as characteristic of Parkinson’s disease, it has some limitations. First, we allowed study patients to continue taking their medications, including dopamine agonists; thus, beta–gamma PAC in the primary motor cortex might have been suppressed in the patients. Dopamine agonists are known to suppress exaggerated PAC in the subthalamic nucleus and the precentral gyrus in patients with Parkinson’s disease^7,34,35,38^. Beta–gamma PAC might therefore be underestimated in our patients with Parkinson’s disease, which might have led to the lack of difference in SI values in the direct comparison between patients and HSPs, as discussed earlier. Additionally, many of the previous studies of PAC and cortical power in Parkinson's disease were conducted under medication withdrawal, making it difficult to perform simple comparisons between those previous findings and our findings obtained in patients who were still taking medication. Second, MEG signals were occasionally contaminated by noise resulting from muscle tension or head movement during recordings. Procedural factors might thus have affected the number of patients who exhibited severe symptoms.

## Conclusions

Our results demonstrated that beta–gamma PAC measured using MEG signals during the resting state is a biomarker for the motor symptoms of Parkinson’s disease. As a noninvasive measure, PAC estimated from MEG signals could be used to help monitor a patient’s symptoms and reveal the pathology behind Parkinson’s disease.

## Methods

### Participants

We recruited patients with Parkinson’s disease from the Neurosurgery and Neurology Departments of Osaka University Hospital, Osaka, Japan. HSPs were recruited through a web page hosted by the university.

Trained neurologists examined all patients with Parkinson’s disease and ensured that they met the Parkinson’s UK Brain Bank criteria^39^. We excluded patients who had essential tremors and limb paralysis because those symptoms could alter the PAC^9^. Trained clinicians used the MDS-UPDRS-III to assess patients’ motor impairments^40^. We did not require patients to stop their medications to participate in the study. The total dose of dopaminergic antiparkinsonian drugs was converted to a levodopa equivalent daily dose^41^.

HSPs had to meet the following inclusion criteria: (1) Japanese ethnicity; (2) 50 years of age or older; (3) no personal or familial diagnostic history of psychiatric disorder and no consultation with a psychiatrist, psychotherapist, neurologist, or neurosurgeon; (4) no personal or familial history of psychotropic drug use, including sleeping pills, anxiolytics, antidepressants, and antipsychotics; (5) no history of oral steroid or immunosuppressant use; (6) no history of syncope; (7) no history of organic cerebral disorders, including brain tumors or strokes; (8) no history of head injuries; (9) no history of meningitis; (10) no history of movement, sensory, or gait disturbances; (11) no history of spinal disease or arthropathy; (12) no history of thyroid disease; (13) no history of hypertension; and (14) no history of chronic liver disease or chronic kidney disease. We excluded participants if they could not remain still during the MEG recording, if they slept during the recording, or if the MEG data could not be acquired because of problems with the machine.

The study adhered to the guidelines set by the Declaration of Helsinki and was performed in accordance with protocols approved by the Research Ethics Committee of the Osaka University Clinical Trial Centre (no. 14448, UMIN000022957). Each participant provided written informed consent after being informed of the study’s purpose. For the patients who were unable to make their own decisions about participation in the study, consent from their legal guardian was requested. However, all patients who participated in this study had normal cognitive and decision abilities and were able to give their own consent for participation.

### MEG data acquisition

MEG data were acquired using a 160-channel whole-head MEG system (MEG Vision NEO, Yokogawa Electric Corporation, Tokyo, Japan). Each participant placed himself or herself in a supine position in a magnetically shielded room, where 5 head-marker coils were attached to his or her face before MEG recording started. Before and after each MEG recording, the positions of the head-marker coils were measured to determine the position and orientation of the MEG sensors relative to the participant’s head. The maximum acceptable difference in position from recording start to recording end was 5 mm. To align MEG data with individual magnetic resonance imaging data (T1 weighted; Signa HDxt Excite 3.0 T, GE Healthcare UK Ltd., Buckinghamshire, UK), we scanned the three-dimensional facial surface and 50 points on each participant’s scalp (FastSCAN Cobra, Polhemus, Colchester, Vermont, USA). The recording-pass band was 0.1–500 Hz, with a sample rate of 2000 Hz.

All participants were instructed to remain awake in a resting state while in the MEG scanner and to keep their eyes closed without thinking about anything in particular. The instruction to keep eyes closed was designed to avoid artifacts from eye blinking. With participants in this state, continuous MEG data were acquired for more than 240 s.

#### Preprocessing of MEG signals

We applied the continuously adjusted least-squares method^42^, performed with MEG Laboratory software (Yokogawa Electric Corporation) using two reference magnetometers, to eliminate environmental (offline) noise. We used Brainstorm^43^ with default parameters for MEG data preprocessing and MEG source imaging.

**Figure 6:**
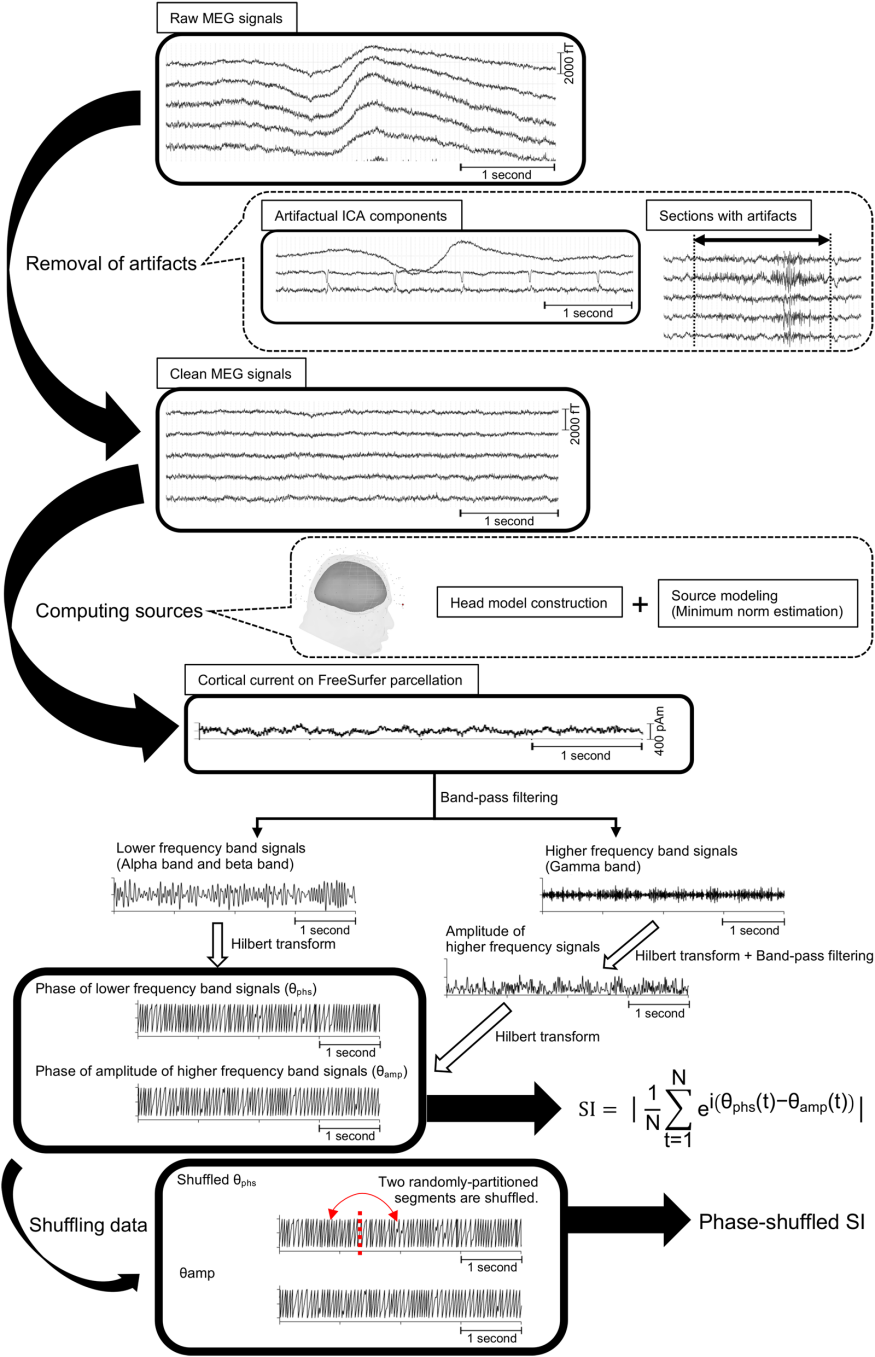
Evaluation of phase-amplitude coupling (PAC) determined from MEG signals.

Of the original 160 channels, we excluded 10 in the temporal region for all participants because those channels were easily contaminated by muscle artifacts. MEG data resampling at 1000 Hz and high-pass filtering at 0.5 Hz were performed for baseline correction. In an independent component analysis, we used the infomax algorithm implemented in Brainstorm to isolate ocular and cardiac artifacts. That algorithm calls the function runica.m from the EEGLAB toolbox^44^. Afterwards, we visually inspected the MEG data to detect remaining segments that were contaminated by muscle artifacts or environmental noise with reference to the records or video images obtained during MEG acquisition. After visually selecting the contaminated segments, we confirmed that the maximum amplitude in the contaminated segments had a high standard deviation (SD), exceeding 5 SDs over the clean segments. Confirmed contaminated segments in all channels were discarded from subsequent analyses (Fig. 6). The length of the data used for each participant in the subsequent analysis is shown in Supplementary Table S1. To eliminate powerline contamination, we applied a band-stop filter at 60 Hz with a width of 1.5 Hz to the clean MEG data. Finally, to increase the calculation speed, the MEG data were resampled at 500 Hz. Data with irremediable artifacts, usually caused by dental implants, were excluded from further analysis.

This process used synchronization index (SI) values to evaluate the PAC and began with an independent component analysis to eliminate artifacts, such as cardiac artifacts and eye blinking, from the raw MEG signals. Subsequently, some noisy epochs were removed. The clean MEG signals were then used to compute the source currents on a cortical surface model. The cortical currents were estimated for each vertex point of the FreeSurfer atlas. The estimated cortical currents were bandpass filtered using 2 different frequency bands: a lower frequency band and a higher frequency band. Hilbert transformation was then used to determine the phase of the lower frequency band signals (θ_phs_) and the amplitude of the higher frequency band signals. Afterwards, the amplitude data from the higher frequency signals were bandpass filtered again by the lower frequency band to determine the amplitude of the higher frequency band signals (θ_amp_). The SI value was calculated using θ_phs_ and θ_amp_. To obtain the shuffled θ_phs_, permuting segments were randomly partitioned at one time point. The phase-shuffled SI was calculated using the shuffled θ_phs_ and θ_amp_. This figure was created using Brainstorm and MATLAB R2015b.

#### Estimation of cortical currents from MEG signals

Information about the scalp and cortical surfaces were extracted from magnetic resonance imaging volume data. For each envelope, we used FreeSurfer software (Martinos Center, Charlestown, Massachusetts, USA)^17^ with default parameter settings to obtain a surface triangulation that was subsequently imported into Brainstorm. The individual high-resolution cortical surfaces (approximately 75,000 vertices per surface) were downsampled to approximately 15,000 triangular vertices (also using a Brainstorm process) to serve as image support for MEG source imaging. We then used the multilinear registration procedure in Brainstorm to transform each vertex location into the FreeSurfer average anatomy^45^ (15,002 vertices). Forward modeling of the neural magnetic fields used the overlapping-sphere technique implemented in Brainstorm^46^. For MEG source imaging, we applied the minimum-norm estimation method to the preprocessed data and estimated the cortical current for each vertex. Further analyses were based on the estimated cortical current.

#### Evaluation of power

The power of the estimated current at each cortical point was calculated for 3 frequency bands (alpha, 8–12 Hz; beta, 13–25 Hz; gamma, 50–100 Hz). For resting-state data, we divided each estimated cortical current into overlapping 1-s time windows shifted by 200 ms. We applied a Hamming window and fast Fourier transform to each time window to obtain a power spectrum. Z score normalization for the whole brain was performed for each frequency band.

The power values at each cortical point were grouped into 360 cortical areas from the Human Connectome Project (HCP) map of cortices^19^ and were averaged for each cortical area.

#### Evaluation of PAC

We used SI^18^ values to evaluate the intensity of the PAC. The SI is defined as.

$$\mathrm{SI}= \left |\frac{1}{{\text{N}}}\sum_{{\text{t}}= \text{1} }^{\text{N}}{{\text{e}}}^{{\text{i}}\left({\theta phs}\text{(}{\text{t}}\text{)-}{\theta amp}\text{(}{\text{t}}\text{)}\right)}\right |,$$

where N is the number of time points in each time window for analysis, θ_phs_*(t)* is the phase value of the lower-frequency-band time series at time point *t,* and θ_amp_*(t)* is the phase value of the fluctuations in the gamma amplitude time series at time *t.* The phase values of the lower frequency band were extracted through Hilbert transformation. The higher frequency band amplitude, filtered by the lower frequency band to obtain phase values, also underwent Hilbert transformation. To calculate the SI values, we subtracted θ_phs_*(t)* and θ_amp_*(t)* of the lower frequency band. That is, the SI values corresponded to the PAC between the higher-frequency-band amplitude and the lower-frequency-band phase. The SI values varied between 0 and 1, with 0 indicating completely desynchronized phases and 1 indicating perfectly synchronized phases.

First, the alpha and beta bands constituted the frequency range of the phase, and the gamma band (50–100 Hz) was the frequency range of the amplitude. SI values based on combinations of these frequencies were calculated on each cortical vertex of the whole brain. Next, to calculate SI values with comprehensive combinations of frequencies in representative cortical areas, the frequency ranges of the phase were 4–8 Hz, 6–10 Hz, 8–12 Hz, 10–14 Hz, 12–16 Hz, 14–18 Hz, 16–20 Hz, 18–22 Hz, and 20–24 Hz; the frequency ranges of the amplitude were 30–80 Hz, 40–90 Hz, 50–100 Hz, 60–110 Hz, 70–120 Hz, 80–130 Hz, 90–140 Hz, and 100–150 Hz. SI values based on all combinations of these frequencies were calculated for representative cortical vertices selected from the left and right frontal regions (the primary motor cortex, HCP parcel #8), the temporal region (anterior area of middle temporal cortex, HCP parcel #134), and the occipital region (the primary visual cortex, HCP parcel #1). After excluding noisy segments, the remaining signals without apparent noise were used as a single time window to evaluate the SI value (Supplementary Table S1). The SI values calculated for each cortical vertex were grouped into 360 cortical areas and averaged for each cortical area.

#### Evaluation of phases

To evaluate the difference in phases for each frequency band for the two participant groups, we defined phase values as follows.

$$\mathrm{Phase\, value \,of \,lower \,frequency \,signals}= \left|\frac{1}{{\text{N}}}\sum_{{\text{t}}= \text{1} }^{\text{N}}{{\text{e}}}^{{\text{i}}\left(\theta \text{phs}\text{(}{\text{t}}\text{)}\right)}\right|,$$

$$\mathrm{Phase\, value \,of \,amplitude \,of \,higher \,frequency \,signals}=\left|\frac{1}{{\text{N}}}\sum_{{\text{t}}= \text{1} }^{\text{N}}{{\text{e}}}^{{\text{i}}\left(\theta \text{amp}\text{(}{\text{t}}\text{)}\right)}\right |,$$

The phase values for each frequency for each cortical vertex were grouped into 360 cortical areas and averaged for each cortical area.

#### Prediction of MDS-UPDRS-III scores from PAC and power

We estimated the predictive capability of both beta–gamma PAC and beta-band power measured during a resting state with respect to the severity of motor impairments in patients with Parkinson's disease. We used MDS-UPDRS-III scores for akinesia to quantify motor impairments, calculated as the sum of 3.4, finger tapping; 3.5, hand movements; 3.6, pronation–supination movements of hands; 3.7, toe tapping; and 3.8, leg agility. We used the area-averaged SI values in the sensorimotor cortex, the temporal cortex and the visual cortex in which significant SI values were present to assess PAC. In the same cortical areas, we used averaged Z scores to assess beta-band power. A Pearson correlation coefficient was used to quantify the relationship between MDS-UPDRS-III scores for akinesia and SI values or Z scores. In the same manner, correlation coefficients were calculated for the left frontal cortex, where beta-band power was observed to be significantly higher in the patients with Parkinson's disease than in HSPs.

#### Evaluation of the relationship between MEG data length and PAC

To evaluate the relationship between MEG data length and PAC, the Pearson correlation coefficient was calculated between data length and SI values for alpha–gamma or beta–gamma PAC averaged over each participant’s entire cortical area.

#### Evaluation of the relationship between PAC and power

To assess the extent to which PAC is influenced by power, the Pearson correlation coefficient was calculated between beta–gamma SI values and the Z score for beta-band or gamma-band power in each cortical area.

### Statistical analysis

Statistical analyses were performed in MATLAB R2015b software (MathWorks, Natick, Massachusetts, USA) and MNE software^47^.

To determine whether the SI values were statistically significant, we calculated SI values using phase-shuffled data, in which the time series of the low-frequency phase was shuffled by permuting segments that were randomly partitioned at one time point^48,49^. We calculated 1000 phase-shuffled SI values for 1 SI value and averaged these phase-shuffled values. By using a paired *t test* to compare the SI values with the mean phase-shuffled SI values for the patient group and the HSP group, a cortical area with a significant SI value was determined for each participant group. To control for false discovery rates across cortical areas and combinations of frequencies, we calculated corrected *P* values at each cortical area by comparing the distribution of *t* values with the distribution of permutated *t* values expected by chance.

To compare the SI values, Z scores for power, and the phase values of each cortical area for the two participant groups, cluster-based permutation *F test*s were performed^50^. All analyses were 2-sided, with a significance level of 0.05.

We performed permutation testing to determine the significance of correlations between MDS-UPDRS-III scores for akinesia and the PAC or between MDS-UPDRS-III scores for akinesia and power; more specifically, we evaluated whether more or fewer significant correlations were observed than would be expected by chance. SI values and Z scores were randomly permuted for participants, while the MDS-UPDRS-III scores were kept the same. We computed a surrogate correlation coefficient based on those shuffled data and repeated the procedure for 2000 permutations, yielding a null distribution of numbers of significant correlations. Significant correlation coefficients were then defined as those that differed significantly from the null distribution.

The number of patients included in the study was calculated in an a priori power analysis in the G*Power program^51^. Our null hypothesis—that the correlation coefficients would differ from 0 in the negative direction—was based on previous studies^12,38^. The effect size (ρ), power to be achieved (β), and probability of an alpha error were set to 0.60, 0.80, and 0.05, respectively. We determined the sample size to be 30, with an expected loss of 30%.

## Supplementary Information

Supplementary Information.

## Data Availability

The data that support the findings of this study are openly available at http://doi.org/10.6084/m9.figshare.12986111.
