# Supplementary material for: Magnetoencephalography detects phase-amplitude coupling in Parkinson’s disease
Source: Sci Rep. 2022 Feb 3;12:1835. doi: 10.1038/s41598-022-05901-9 (PMC8813926; doi:10.1038/s41598-022-05901-9)
Supplement: Supplementary file 1 — Supplementary Information. [file 41598_2022_5901_MOESM1_ESM.pdf]

Supplementary information for

Magnetoencephalography detects phase-amplitude coupling in Parkinson's disease

Authors:

Masataka Tanaka,[1] Takufumi Yanagisawa,[1–3*] Ryohei Fukuma,[1,3] Naoki Tani,[1] Satoru Oshino,[1] Masahito Mihara,[4] Noriaki Hattori,[4,5] Yuta Kajiyama,[4] Ryota Hashimoto,[6–8] Manabu Ikeda,[7] Hideki Mochizuki,[4] Haruhiko Kishima[1]

Affiliations:

[1]Department of Neurosurgery, Osaka University Graduate School of Medicine, 2-2 Yamadaoka, Suita, Osaka 565-0871 Japan

[2]Institute for Advanced Co-Creation Studies, Osaka University, 2-2 Yamadaoka, Suita, Osaka 565-0871 Japan

[3]ATR Computational Neuroscience Laboratories, Department of Neuroinformatics, 2-2-2 Hikaridai, Seika-cho, Kyoto 619 0288 Japan

[4]Department of Neurology, Osaka University Graduate School of Medicine, 2-2 Yamadaoka, Suita, Osaka 565-0871 Japan

[5]Department of Rehabilitation, Faculty of Medicine, Academic Assembly, University of Toyama

[6]Department of Pathology of Mental Diseases, National Institute of Mental Health, National Center of Neurology and Psychiatry, 4-1 Ogawahigashi, Kodaira, Tokyo 187-8553 Japan

[7]Department of Psychiatry, Osaka University Graduate School of Medicine, 2-2 Yamadaoka, Suita, Osaka 565-0871 Japan

[8]Molecular Research Center for Children's Mental Development, United Graduate School of Child Development, Osaka University, 2-2 Yamadaoka, Suita, Osaka 565-0871 Japan

Table S1. Magnetoencephalography data length used in the analysis

Patient ID	MEG data length (s)	HSP ID	MEG data length (s)
1	232.5	1	235.9
2	236.0	2	235.5
3	236.0	3	234.4
4	236.0	4	234.3
5	235.4	5	229.7
6	234.6	6	236.0
7	236.0	7	236.0
8	226.7	8	236.0
9	229.1	9	236.0
10	235.1	10	235.0
11	235.3	11	233.7
12	236.0	12	235.4
13	235.2	13	234.1
14	236.0	14	234.1
15	236.0	15	236.0
16	235.1	16	233.8
17	229.2	17	236.0
18	235.5	18	235.4
19	235.8	19	227.7
20	231.0	20	236.0
21	234.4	21	236.0
22	236.0	22	232.1
23	235.5	23	236.0

MEG, magnetoencephalography; HSP, healthy study participant

Here, we were concerned that excluding noisy magnetoencephalography (MEG) segments from our analysis would cause differences in the data length between the participant groups, which could affect the results of the analysis. Although we removed the noisy epochs for each participant, the mean length of the MEG data used in the analysis was not significantly different between the two groups: 234.2 ± 2.7 s for patients with Parkinson's disease and 234.6 ± 2.1 s for healthy study participants (HSPs). In addition, no significant correlations were evident between data length and the synchronization index (SI) values averaged over the entire cortical area (alpha–gamma phase–amplitude coupling [PAC], $r = 0.15$, $P = 0.26$; beta–gamma PAC,

$r = -0.05$, $P = 0.63$), suggesting that removal of the noisy signals did not significantly affect the results from the SI values.

Table S2. Areas of the brain defined by the HCP atlas where significant beta phase–gamma amplitude coupling was present

Participant group	Section	Parcel index and area description
Patients	1 (Early visual cortex)	**L** 181 (Primary visual cortex)
	2 (Dorsal stream)	**L** 4 (Second visual area), 5 (Third visual area), 6 (Fourth visual area)<br>**R** 184 (Second visual area), 185 (Third visual area), 186 (Fourth visual area)
	4 (MT+ complex)	**L** 7 (Eighth visual area), 18 (Fusiform face complex), 22 (Posterior inferotemporal), 154 (Ventromedial visual area 3), 163 (Ventral visual complex)<br>**R** 198 (Fusiform face complex), 333 (Ventromedial visual area 1), 343 (Ventral visual complex)
	5 (Neighbors)	**L** 138 (Area PH)<br>**R** 182 (Medial superior temporal area)
	6 (Early somatosensory and motor cortex)	**L** 8 (Primary motor cortex), 9 (Primary sensory cortex), 53 (Area 3a)<br>**R** 188 (Primary motor cortex), 233 (Area 3a)
	7 (Sensorimotor-associated paracentral lobular and)	**R** 220 (Dorsal area 24d)
	9 (Posterior opercular cortex)	**R** 282 (Area OP2-3/vs.)
	11 (Association auditory cortex)	**R** 303 (Area STGa), 308 (Area STSd anterior)
	12 (Insular and frontal opercular cortex)	**L** 110 (Pirform cortex)<br>**R** 288 (Frontal opercular area 4), 290 (Pirform cortex)

	13 (Remaining areas of the temporal cortex including the medial area)	**L** 118 (Entorhinal cortex), 119 (Presubiculum), 120 (Hippocampus), 122 (Perirhinal ectorhinal cortex), 126 (Parahippocampal area 1), 135 (Area TF), 155 (Parahippocampal area 2)<br>**R** 298 (Entorhinal cortex), 299 (Presubiculum), 302 (Perirhinal ectorhinal cortex), 306 (Parahippocampal area 1), 307 (Parahippocampal area 3), 315 (Area TF), 335 (Parahippocampal area 2)
	14 (Lateral temporal cortex)	**L** 131 (Area TG dorsal), 134 (Area TE2 anterior)<br>**R** 314 (Area TE2 anterior), 316 (Area TE2 posterior),352 (Area TG ventral)
	20 (Orbital and polar frontal cortex)	**L** 92 (Area 13l)<br>**R** 274 (Area 47s)
	21 (Inferior frontal cortex)	**R** 256 (Area 47 lateral), 262 (Area IFSa), 351 (Area posterior 47r)
HSPs	6 (Early somatosensory and motor cortex)	**L** 9 (Primary sensory cortex)<br>**R** 232 (Area 2)
	7 (Sensorimotor-associated paracentral lobular and mid cingulate cortex)	**R** 219 (Area 5L)
	13 (Remaining areas of the temporal cortex including the medial area)	**R** 298 (Entorhinal cortex), 302 (Perirhinal ectorhinal cortex), 307 (Parahippocampal area 3)

HCP, Human Connectome Project; HSP, healthy study participant; L, left; PAC, phase–amplitude coupling; R, right.

Figure S1. Phase values during the resting state.

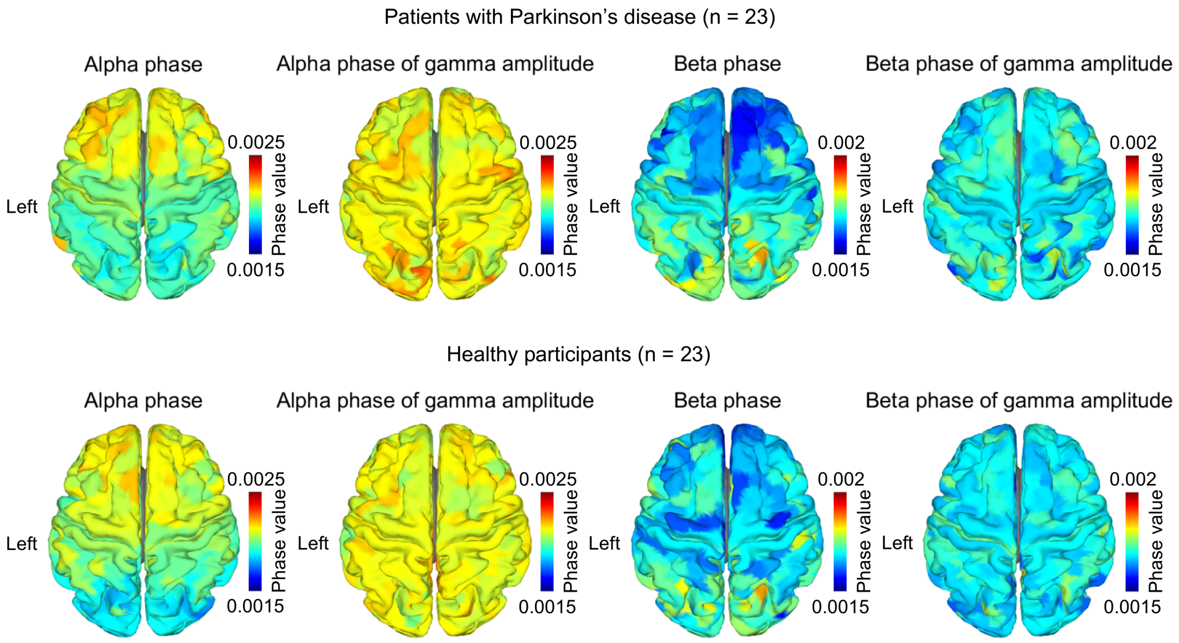

For each participant group (patients with Parkinson's disease and healthy study participants), we calculated the complex exponential of the analytic phase to obtain the absolute value of the mean for the lower frequency bands (alpha and beta) and the gamma-band amplitude filtered by the lower frequency bands. The absolute values of the mean (labeled as phase values) were color-coded on the cortices of patients (n = 23) and healthy participants (n = 23). No statistically significant differences were observed between the patients and the healthy participants.

Figure S2. Correlations between disease symptoms and PAC or between disease symptoms and power in the temporal cortex, the visual cortex and the left frontal cortex.

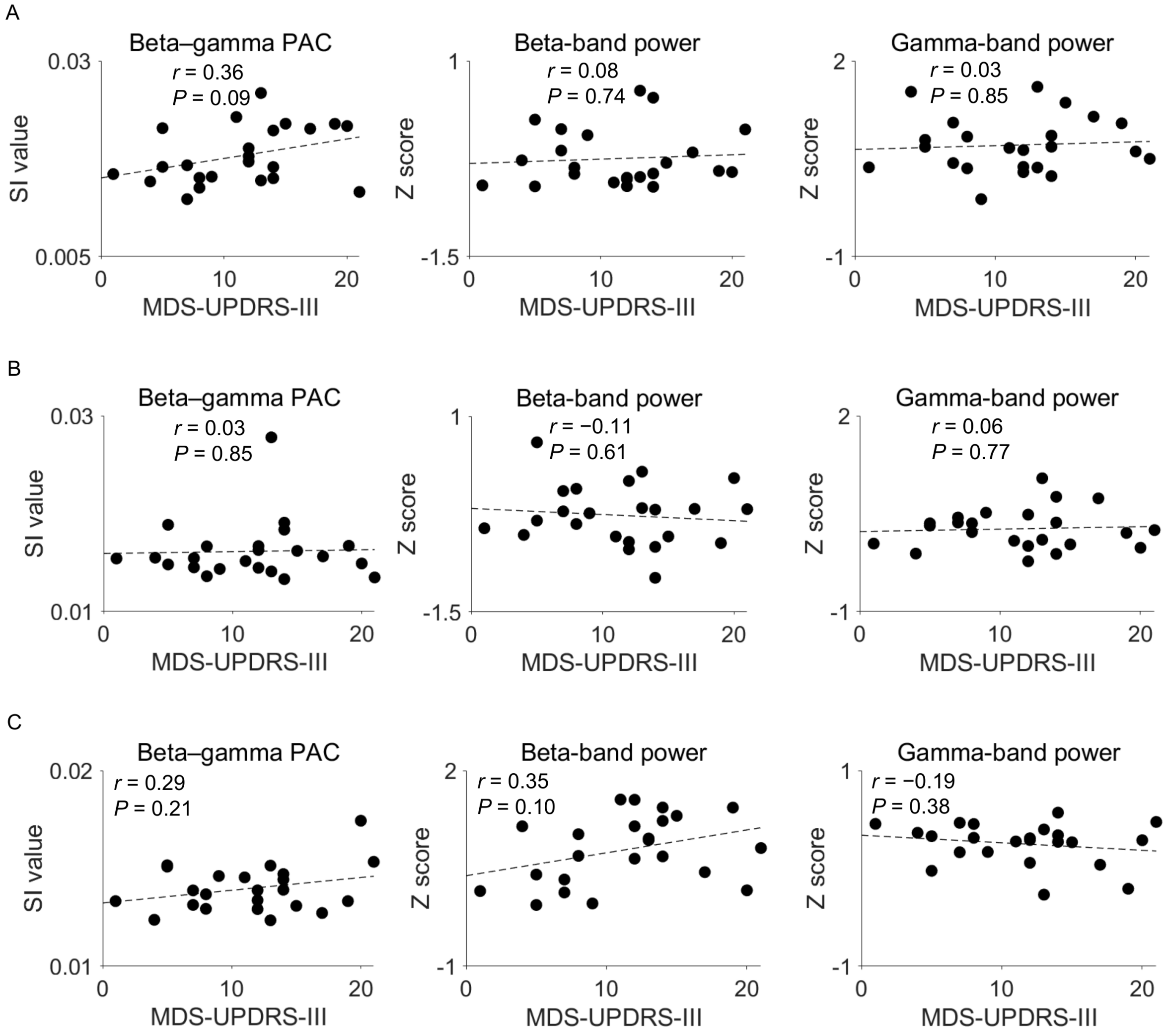

Correlations were evaluated between disease symptoms and beta–gamma phase–amplitude coupling, between disease symptoms and beta-band power or between disease symptoms and gamma-band power in the temporal cortex (A), the visual cortex (B) and the left frontal cortex (C). The plots on the left show the correlation between averaged synchronization index values for the temporal cortex, the visual cortex or the left frontal cortex and the sum of the MDS-UPDRS-III scores for akinesia. The plots in the center and the ones on the right show correlations between the averaged Z scores for the temporal cortex, the visual cortex or the left frontal cortex and the sum of the MDS-UPDRS-III scores for akinesia. In the plots, each dot represents a patient with Parkinson's disease (n = 23); dashed least-squares lines are also shown. MDS-UPDRS-III = Movement Disorder Society–sponsored revision of the Unified Parkinson's Disease Rating Scale, part III.

Figure S3. Correlations between power and PAC.

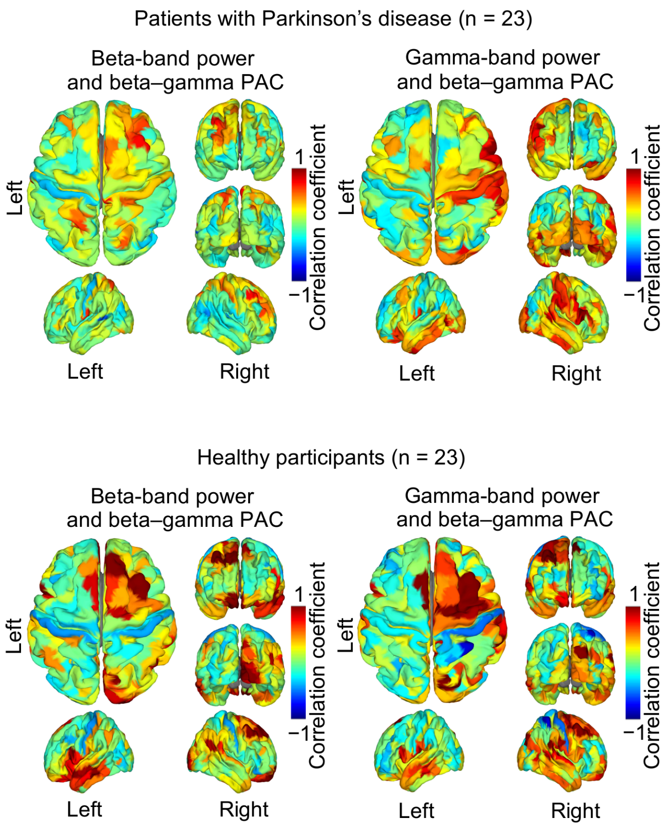

Correlations between Z score powers and the SI values for PAC were evaluated. Pearson correlation coefficients were calculated between Z scores for beta-band power and the SI values for beta–gamma PAC or between gamma-band power and the SI values for beta–gamma PAC in each cortical area for each participant group; Z scores were color-coded on the cortex. PAC = phase–amplitude coupling; SI = synchronization index;
